# The role of CB_2_ receptor ligands in human eosinophil function

**DOI:** 10.1186/2050-6511-13-S1-A13

**Published:** 2012-09-17

**Authors:** Robert Frei, Eva Sturm, Ákos Heinemann

**Affiliations:** 1 Institute of Experimental and Clinical Pharmacology, Medical University of Graz, 8010 Graz, Austria

## Background

Eosinophils play a key role in allergic diseases such as bronchial asthma and atopic dermatitis. A prominent feature of these diseases is the accumulation of eosinophils in inflamed tissue induced by several chemoattractants like prostaglandin (PG) D_2_ or eotaxins. After the discovery of the endocannabinoid system and investigation of several endogenous and synthetic ligands, evidence has accumulated that cannabinoids, especially CB_2_ receptor ligands, may play a major role in mediating inflammatory responses. Elevated levels of 2-arachidonoylglycerol (2-AG; a CB_1_/CB_2_ agonist) were found in tissues of mouse models of allergic inflammation, suggesting a possible involvement in leukocyte recruitment.

## Methods

Blood was sampled from healthy volunteers, erythrocytes were removed by dextran sedimentation and polymorphonuclear leukocytes were obtained via Histopaque gradients. For all assays eosinophils were further purified by negative magnetic isolation. Shape change was recorded immediately after stimulation on a FACSCalibur flow cytometer. For chemotaxis assays an AP48 microBoyden chamber was used and migration time was 1h at 37°C. Intracellular Ca^2+^ levels were analyzed by flow cytometry after treating eosinophils with Fluo-3-AM for 60min at room temperature.

## Results

We found that CB_2_ receptor agonists like the endocannabinoid 2-AG or the synthetic selective agonist JWH-133 significantly increased eosinophil responses in shape change assays induced by PGD_2_ or eotaxin-1. The observed effects could be abolished by pretreatment with the selective CB_2_ receptor antagonists SR144528 or AM630. As cytoskeletal changes are required for firm arrest and leukocyte diapedesis, transmigration assays were conducted subsequently, confirming the shape change data as eosinophil migration induced by PGD_2_ was increased by pretreatment with JWH-133. Calcium flux assays showed Ca^2+^ mobilization induced by JWH-133 and the tendency to increase PGD_2_-induced Ca^2+^ release.

## Conclusions

We could show that JWH-133 can influence human eosinophil activation/migration via activation of CB_2_ receptors as chemoattractant effects were significantly modulated, suggesting a possible pro-inflammatory role in allergic inflammation which may further lead to cannabinoid-based treatment options in allergic inflammatory diseases.

